# Epidural Spinal Cord Stimulation Facilitates Immediate Restoration of Dormant Motor and Autonomic Supraspinal Pathways after Chronic Neurologically Complete Spinal Cord Injury

**DOI:** 10.1089/neu.2018.6006

**Published:** 2019-07-12

**Authors:** David Darrow, David Balser, Theoden I. Netoff, Andrei Krassioukov, Aaron Phillips, Ann Parr, Uzma Samadani

**Affiliations:** ^1^ Department of Neurosurgery, University of Minnesota, Minneapolis, Minnesota.; ^2^ Department of Surgery, Division of Neurosurgery, Minneapolis VA Health Care System, Minneapolis, Minnesota.; ^3^ Department of Biomedical Engineering, University of Minnesota, Minneapolis, Minnesota.; ^4^ International Collaboration on Repair Discoveries; Division of Physical Medicine and Rehabilitation, University of British Columbia, Vancouver, British Columbia, Canada.; ^5^ Departments of Physiology and Pharmacology, Cumming School of Medicine, University of Calgary, Calgary, Alberta, Canada.; ^6^ Department of Neurosurgery, Hennepin County Medical Center, Minneapolis, Minnesota.

**Keywords:** autonomic, blood pressure, cardiovascular dysautonomia, spinal cord injury, spinal cord stimulation

## Abstract

Epidural Spinal Cord Stimulation (eSCS) in combination with extensive rehabilitation has been reported to restore volitional movement in a select group of subjects after motor-complete spinal cord injury (SCI). Numerous questions about the generalizability of these findings to patients with longer term SCI have arisen, especially regarding the possibility of restoring autonomic function. To better understand the effect of eSCS on volitional movement and autonomic function, two female participants five and 10 years after injury at ages 48 and 52, respectively, with minimal spinal cord preservation on magnetic resonance imaging were implanted with an eSCS system at the vertebral T12 level. We demonstrated that eSCS can restore volitional movement immediately in two female participants in their fifth and sixth decade of life with motor and sensory-complete SCI, five and 10 years after sustaining severe radiographic injuries, and without prescribed or significant pre-habilitation. Both patients experienced significant improvements in surface electromyography power during a volitional control task with eSCS on. Cardiovascular function was also restored with eSCS in one participant with cardiovascular dysautonomia using specific eSCS settings during tilt challenge while not affecting function in a participant with normal cardiovascular function. Orgasm was achieved for the first time since injury in one participant with and immediately after eSCS. Bowel-bladder synergy improved in both participants while restoring volitional urination in one with eSCS. While numerous questions remain, the ability to restore some supraspinal control over motor function below the level of injury, cardiovascular function, sexual function, and bowel and bladder function should promote intense efforts to investigate and develop optimization strategies to maximize recovery in all participants with chronic SCI.

## Introduction

Despite more than five decades of clinical trials and research, there have been few major advancements in the clinical treatment of persons with chronic spinal cord injury (SCI). Spinal cord stimulation (SCS)—and more specifically, epidural SCS (eSCS)—involves the surgical implantation of a small array of electrodes into the epidural space, originally developed to manage chronic pain, and has been described since 1967.^1^ After more than 40 years of attempting to use eSCS for patients with SCI,^2^ the possibility of restoring volitional movement below the level of injury in patients with motor-complete SCI was discovered nearly a decade ago.^3–5^ It was serendipitously discovered that the restoration of volitional movement (supraspinal control) was possible after motor-complete SCI.^5^

A limited number of highly publicized reports^5–9^ have shown that carefully selected male participants (and more recently, one 22-year-old woman three years from injury)^10^ with motor-complete SCI (American Spinal Injury Association Impairment Scale [AIS] A and B)^11^ coupled with long-term intensive locomotor training can restore supraspinal control over spinal cord motor circuits through eSCS applied below the level of injury.^3,4^ It was suggested that intensive training may be crucial to reactivate and form new supraspinal connections that allowed for eSCS to facilitate voluntary movement, which has been strongly supported in animal studies using causal techniques and sophisticated imaging of the specific pathways.^5,12^

After these initial reports, questions arose regarding the ability to generalize this therapy to the broader SCI population.^13^ A key first question is whether radiographically severe injuries would obviate meaningful restoration of function with eSCS. Numerous mechanisms underlying the observed motor recovery during eSCS have been proposed and include large diameter afferent activation leading to increased net excitability of caudal circuits, recruitment of spared descending fibers, and potentially segmental disinhibition.^14^ Based on these hypotheses, partial sparing of descending tracts through the injured spinal cord remains critical, but the most minimal amount of axonal preservation to achieve meaningful functional improvement with eSCS has not been determined.

A second key question is whether eSCS can improve function in participants with long-term chronic SCI, who are typically at more advanced ages. The average time from injury in the general population is close to 20 years^15^; however, the maximum time since injury in the currently reported eSCS studies for SCI is four years with a maximum age of 32 years.^5–9^ A third key question is whether the pattern of recovery is consistent and predictable when comparing individuals. Given the complexity of the spinal cord and target muscles, systematic and quantitative assessment of patterns of recovery with useful summary measures may facilitate more generalizable results across trials with the goal of refining patient selection and expectations as well as designing optimal rehabilitation solutions.

A final key question, which remains of significant interest to individuals with SCI,^16–18^ is whether eSCS can restore autonomic control of cardiovascular, bowel, bladder, and sexual function. Most recently, studies involving men and women 35 years of age or younger have explored improvements in bowel, bladder, sexual, and cardiovascular function.^19,20^ Further exploration of these findings in a wider demographic in terms of age and years since injury will help determine the potential applicability of this intervention in the general SCI population.

We present the initial findings from the Epidural Stimulation After Neurologic Damage clinical trial (E-STAND, Trial Number: NCT03026816), designed to address these key questions. We designed the E-STAND trial to begin to answer some of these questions by enrolling a generalizable patient population, quantitatively assessing the effects of eSCS on the restoration of volitional muscle activity, and assessing autonomic effects including cardiovascular, bowel, bladder, and sexual function.

Our preliminary findings suggest that eSCS may be beneficial for a dramatically broader group of patients than has been studied previously, including women (only one recently reported),^10^ without intensive pre-implantation protocoled training, with minimal axonal preservation on standard magnetic resonance imaging (MRI), more than 10 years from injury, and patients in the fifth and sixth decade of life. We also report the use of a non-invasive, quantitative method of describing the complex pattern of restoration of volitional movement using surface electromyography (sEMG). Finally, our data indicate that eSCS can also provide benefit for cardiovascular, bowel/bladder, and sexual function.

## Patients and Methods

### Study design and participants

This study was performed in accordance with local Institutional Review Board approval and Food and Drug Administration Investigational Device Exemption approval. The participants identified in this study have provided informed consent and authorization to present publicly identifiable information for research purposes. The study protocol is registered with ClinicalTrials.gov (NCT03026816). Patients with chronic, traumatic SCI (greater than one year since injury) were recruited if they met the following criteria: greater than 22 years of age, motor complete AIS classification A or B with a neurological level of injury between C6 and T10, full arm and hand strength, and intact segmental reflexes below the level of injury.

Participants were excluded if they had medical or psychological comorbidities that would significantly increase the risk of operation, severe dysautonomia with systolic blood pressure fluctuation below 50 or above 200 mmHg on autonomic testing, contractures, pressure ulcers, recurrent urinary tract infection, unhealed spinal fracture, recent botulinum toxin use, or pregnancy. Tilt-table testing performed during screening identified participants with cardiovascular dysautonomia, who were then enrolled into the autonomic arm with the addition of cardiovascular testing. Patients returned for monthly assessments for stimulator programming, surveys, and sEMG Brain Motor Control Assessment (BMCA).^21^

Two female participants with chronic thoracic AIS A SCI were enrolled in the study. Participant 1 was 52 years of age, had a T8 AIS A injury after a fall, and was 10 years from injury. Participant 2 was 48 years of age, had a T4 AIS A injury after a motorcycle accident, and was five years from injury. Neither participant had undergone extensive exercise training for the six months before enrolling in the study. Participant 1 did not undergo any home, inpatient, or outpatient rehabilitation in the six months before study participation. Participant 2 reported 60 h of individual outpatient rehabilitation in the six months before study participation including standing and functional electrical stimulation exercises.

### Surgery

Participants underwent implantation of a three column (5-6-5), 16-contact paddle lead with a primary cell internal pulse generator (IPG) (Tripole™ and Proclaim Elite™, Abbott, Plano, TX) at approximately the vertebral T12 level under general anesthesia (Supplementary Fig. 1). Low frequency (2 Hz, 450 μs, 0-10 mA) stimulator-evoked motor responses allowed electrophysiological mapping to ensure coverage of the lumbar and sacral roots (vastus medialis, adductor longus, vastus lateralis, biceps femoris, tibialis anterior, gastrocnemius, rectal sphincter). These muscles were selected to represent major innervation from each nerve root between L2 and S2 according to intraoperative monitoring practice convention at the study institution before the publication of a standard muscle list and procedure.^22^

The paddle was configured with cathodes inferiorly and anodes superiorly. The paddle was shifted intraoperatively until maximal and symmetric muscle recruitment was observed with the most minimal current. Anchors were placed and sutured to secure the paddle, and the wires from the paddle electrode were tunneled to a subcutaneous pocket where the pulse generator was secured. Surgery was performed on an outpatient basis with participants leaving the hospital the same day.

### Assessment of volitional muscle activity

A Natus Nicolet Electrodiagnostic system with an Endeavor sixteen-channel intraoperative module (Natus Medical Incorporated, Pleasanton, CA) was used for sEMG recordings. Each participant underwent two surface sEMG assessments at enrollment and at each monthly visit: once with stimulation on and once with stimulation off. The SCS stimulator settings were set before beginning assessment and remained unchanged throughout.

Eight total muscle groups (rectus abdominis, T10 intercostal, T12 paraspinal, iliopsoas, rectus femoris, tibialis anterior, extensor hallucis longus, and gastrocnemius) on each side were recorded (five in the lower extremities) while the subject lying supine with enough head elevation so that she could observe her legs. The selected muscles representing each nerve root differed from the ones utilized during intraoperative monitoring because of the differences in anatomical accessibility when comparing needle and surface monitoring. A modified version of the BMCA was used, where volitional tasks included six maneuvers performed three times (bilateral and unilateral hip flexion/extension with knee flexion/extension, as well as bilateral and unilateral dorsiflexion/plantarflexion). A five-minute resting phase was performed during the task to differentiate involuntary and evoked movements from volitional movements.

Recordings of the 10 lower extremity muscle groups during each of these maneuvers, averaged over three trials, formed the basis for further evaluation. The average root mean square (RMS) surface EMG power was calculated for each task and muscle group (labeled as Raw in Supplementary Fig. 2). Surface EMG recordings are inherently noisy. Baseline noise present on each channel was calculated as the RMS of the last 5 sec for each trial and channel. Surface EMG power (referred to as power for the remainder of the article) from noise was subtracted from raw power to identify power from muscle activity (Raw - Noise in Supplementary Fig. 2).

To account for movements from stimulation without intention or volition, power was calculated for each channel over 30 windows of 10 sec during a 5 min relaxation period. The median power of each channel was used to calculate an evoked power. This power was subtracted to produce the Raw - Noise Response Magnitude (modified from previous reports),^23^ labeled as Nonstimulation or Volitional (Stim) in Figure 3. Further details are available in the Supplementary Text.

### Experimental stimulation

The array of stimulator electrodes was programmed to stimulate broadly and adjusted to provide symmetrical responses. While the frequency (16–400 Hz) and pulse width (200–500 μs) were varied as part of the optimization component of the study, the current was adjusted to allow a range, where the bottom of the range was found by identifying signs of movement and the midpoint of the range was programmed to be maximal volitional movement while supine. Outside of monthly testing, patients were free to adjust stimulation intensity to compensate for impedance changes in different positions (e.g., supine vs. upright). As a result, amplitude varied from a minimum of 2 mA to 15 mA, depending on position.

Stimulation for autonomic function (CV Stim) used an electrode configuration that maximized the current density above the electrode, rostrally, with the goal of exciting some of the caudal pre-ganglionic neurons, which are distributed between T1 and L2. Because the study was designed to systematically evaluate the effect of various eSCS parameter settings on volitional movement, parameter settings used for the primary outcome varied considerably as a result of using the best of eight settings assigned the previous month to evaluate the large parameter space during the month between visits. Further information is available in the Supplement.

### Ambulatory blood pressure (BP) assessments

Participants underwent ambulatory BP monitoring (ABPM-05, Meditech Ltd., Budapest, Hungary) pre-programmed to obtain BP measurements every 15 min during daytime and every 30 min during sleep hours to identify cardiovascular dysautonomia (orthostatic hypotension or autonomic dysreflexia) off stimulation. The cuff was applied by a physician, and participants were instructed to leave it on for 24 h. All participants were screened for signs of cardiovascular dysautonomia during a 10 min tilt-table test that determined eligibility and enrollment in the autonomic arm of the study.

### Hemodynamic assessments

During orthostatic challenge testing (70 degrees tilt-table test), BP and heart rate were measured non-invasively every minute (Suresigns VSi, Koninklijke Philips N.V., Amsterdam, The Netherlands) on the right brachial artery and a beat-by-beat basis via finger photoplethysmography (Finometer PRO, Finapres Medical Systems, Amsterdam, The Netherlands). Electrocardiography (ML 132; ADInstruments) and BP were sampled at 1000 Hz using an analog-to-digital converter (Powerlab/16SP ML 795; ADInstruments, CO Springs, CO) interfaced with data acquisition software on a laptop computer (LabChart 7 ADInstruments). Stroke volume and cardiac contractility (dP/dT) were computed using Modelflow. Further, blood velocity was measured in either the left or right middle cerebral artery, depending on signal quality (Spencer Technology, Redmond, WA), using a 2 MHz probe mounted on the temporal bone and a fitted head strap, according to our described protocol.^24^

Participants rested supine for approximately 10 min while baseline hemodynamic data were recorded. Participants were then tilted to 70 degrees and asked about the presence of pre-syncopal symptoms (i.e., dizziness, lightheadedness, nausea) at the beginning, middle, and end of each stage. All cardiovascular parameters were measured continuously during this test. Once hypotensive, caudal stimulation (used for volitional movement) was applied followed by rostral stimulation for comparison. Cognitive assessments (described below) were performed during baseline, symptomatic tilt, and stimulation.

### Cognitive function measures

Cognitive assessments were performed by an experienced researcher, AP. The following cognitive tests were performed according to standard protocols: Forward and Backward Digit Span Test,^25^ Stroop Test,^26^ and Controlled Oral Word Association Test.^27^

### Bowel and bladder assessments

The Neurogenic Bowel Dysfunction Score (NBDS)^28^ was used to assess bowel function. Summary scores are categorized as very minor, minor, moderate, and severe. The Neurogenic Bladder Symptom Score (NBSS)^29^ was used to assess bladder function after SCI. Scores were divided into the domains of incontinence, consequences, storage, and quality of life.

### Statistical methods

Two sample *t* tests were performed on baseline (non-stimulation), stimulation power, each BMCA maneuver, as well as for systolic BPs obtained at baseline, during symptomatic hypotension, and during stimulation. Normality was evaluated, and log transform was used where appropriate to improve normality. See Supplement for more information; see online supplementary material at www.liebertpub.com. Differences were considered to be statistically significant when *p* < 0.05. Relaxation data during the first appointment of the first patient were imputed from the subsequent four visits because of technical error.

### Role of the funding source

This is an independent investigator-sponsored study. None of the funding sources were involved in data collection, analysis, manuscript writing, or the decision to submit the manuscript for publication. The corresponding author maintains full access to all study data and has the final responsibility in the decision to submit the manuscript.

## Results

### Study participants

**FIG. 1.**
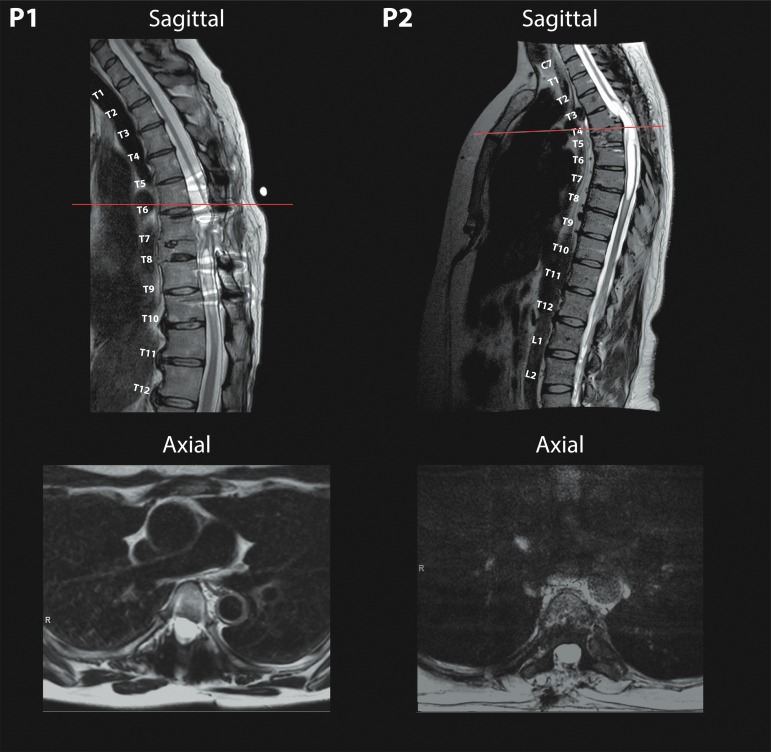
Severe spinal cord injury with associated myelomalacia in both Participant 1 (left) and Participant 2 (right) from T8 and T4/5 injuries, respectively, in midsagittal planes of T2-weighted pre-operative magnetic resonance imaging. Axial slices (bottom) demonstrate minimal remaining spinal cord tissue with corresponding scout lines present on sagittal planes.

Both participants have severe SCI with less than 1 mm of residual cord at the level of the lesion and extensive myelomalacia as revealed by MRI and shown in Figure 1.

### Stimulator implantation

Epidural spinal cord stimulator placement in both participants was guided by intraoperative neuromonitoring. The stimulator paddle in Participant 1 was placed with the leads in the caudal orientation. Final lead placement after stimulator repositioning resulted in EMG response in muscles corresponding to the left L1, L2, L4, L5, S1, and S2 and right L1–L4, S1, and S2 with 2 Hz, 7 mA stimulation. The stimulator paddle in Participant 2 was placed initially with the leads in the caudal direction with limited response in muscles corresponding to L1–L2. The stimulator was inverted (leads in the rostral direction) to better secure the paddle, resulting in EMG responses seen in muscles corresponding to right L1–S2 and left L1–L5 and S2 with 2 Hz, 5 mA stimulation.

### Volitional movements and muscle activity

**FIG. 2.**
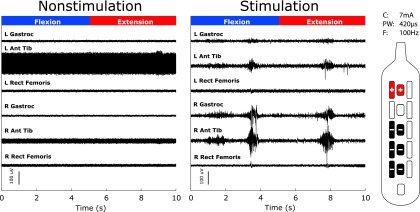
Surface electromyography (sEMG) during volitional tasks. Left panel shows raw baseline surface EMG during the bilateral ankle flexion and extension maneuver with spinal cord stimulation (SCS) off, and the right panel with SCS on. Appearance of large excursions in sEMG activity reflect voluntarily production of muscle contraction in response to verbal command (Participant 2). We observed recruitment of bilateral gastrocnemius and anterior tibialis without recruitment of the rectus femoris. The electrode configuration is shown to the right of the figure. Current (C) 7 mA, pulse-width (PW) 420 μs, and frequency (F) 100 Hz.

**FIG. 3.**
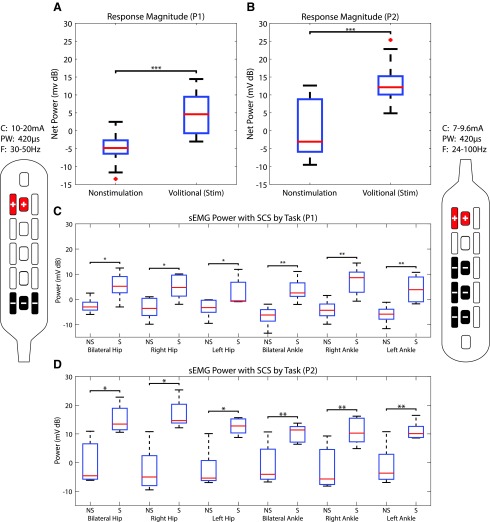
Differences in surface electromyography (sEMG) power. Pooled sEMG power between non-stimulation (NS) and stimulation (S) for the first five follow-up appointments for Participant 1 (P1) (**A**) and Participant 2 (P2) (**B**) demonstrating the power generated during volitional movement during Non-stimulation and stimulation (Volitional [stim]). The sEMG power for each maneuver during the first five follow-up appointments during NS and S (at intervals of approximately one month) for P1 (**C**) and P2 (**D**). Stimulator patterns for P1 are shown on the left, and patterns for P2 are shown on the right. In order of visit, P1 used frequencies (F) of 30, 30, 24, 50, and 30 Hz. Pulse-widths (PW) were 420 μs, and currents (C) were 13.5, 16.5, 10, 20, and 16 mA. P2 used frequencies of 30, 100, 30, 40, and 24 Hz. Pulse-width was 420 μs, and currents used were 7.8, 7, 7.5, 7.8, and 9.6 mA. Outliers (>2.7□) are denoted as red crosses. Asterisks denote statistical significance: *p < 0.05, **p < 0.01, ***p < 0.001.

Voluntary movements of the lower extremities were observed in both participants on the first instance of activating eSCS (example movements are in Supplementary Videos 1 and 2). Acute application of stimulation allowed participants to volitionally activate some muscle groups on command. Raw traces of sEMG during a single trial of plantarflexion and dorsiflexion are demonstrated for Participant 2 in Figure 2. Neither patient had sensation, and observed voluntary activity was found to be coarse and often bilateral with asymmetry in recorded power. Significant improvements in the power during the BMCA with eSCS were measured in both participants, as shown in Figure 3 where non-stimulation is compared with power caused by volitional (Volitional [stim]) power estimated by subtracting background noise and spontaneous muscle activity generated during a 5-min relaxation phase from the raw power.

Participant 1 had a significant improvement in the overall power (*p* < 0.001, two sample *t* test) when compared with non-stimulation/baseline, pooled across trials and maneuvers. Participant 2 had a larger improvement in power (*p* < 0.001, two sample *t* test) with more overall variation. When broken down by individual maneuvers or tasks of the BMCA, Participant 1 demonstrated a significant increase in power across most maneuvers (Fig. 3, panel C) (*p* values: BL Hip (0.01), R Hip (0.01), L Hip (0.04), BL Ankle (0.005), R Ankle (0.003), and L Ankle (0.004). Participant 2 also demonstrated statistically significant improvements in power across maneuvers (Fig. 3, panel D) (*p* values: BL Hip (0.008), R Hip (0.006), L Hip (0.01), BL Ankle (0.02), R Ankle (0.01), and L Ankle (0.02). Power averaged over visits for each muscle group and task during non-stimulation and stimulation BMCA are reported in the Supplement (Supplementary Fig. 3).

### Cardiovascular dysautonomia pre-implantation screening

**FIG. 4.**
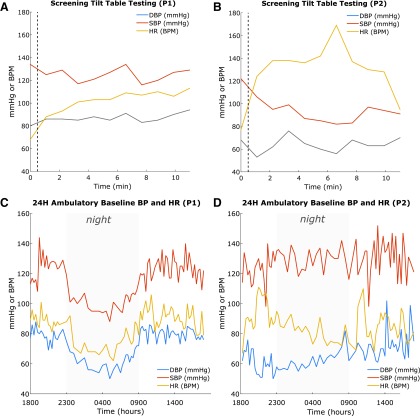
Cardiovascular screening. Top: Tilt-table testing at screening for Participant 1 (P1) (**A**) and Participant 2 (P2) (**B**). The vertical dotted line indicates the time of tilt preceding the 10 min period. Notice the relative stability in P1 (**A**) compared with P2 (**B**). Bottom: Baseline ambulatory blood pressure and heart rate monitoring for 24 h for P1 (**C**) and P2 (**D**) with nocturnal hours denoted by gray. Notice the significant difference between P1 (C), who exhibits normal diurnal variation, and P2 (D), who exhibits little diurnal variation. DBP, diastolic blood pressure; SBP, systolic blood pressure; HR, heart rate.

During pre-operative screening tilt-table testing to 70 degrees, Participant 1 exhibited minimal dysautonomia while Participant 2 demonstrated significant cardiovascular dysfunction (Fig. 4 A,B). During baseline ambulatory BP screening of Participant 1, the 24 h BP showed a normal nocturnal pattern of decreased BP and heart rate (Fig. 4 C) while the hemodynamics of Participant 2 did not vary between diurnal and nocturnal periods (Fig. 4 B), indicating poor autonomic control.

### Stimulation-enabled recovery of cardiovascular function during tilt-table testing

**FIG. 5.**
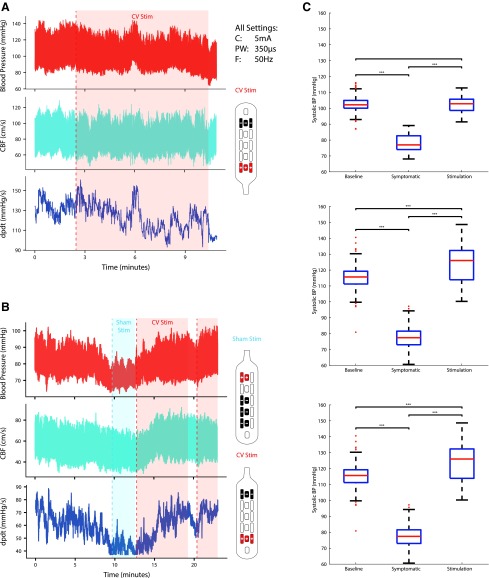
Effect of spinal cord stimulation during tilt. (**A**) Tilt-table testing and stimulation had little effect on blood pressure (BP) for Participant 1 using stimulation for autonomic function (CV Stim). (**B**) Severe orthostatic hypotension during tilt-table evaluation in Participant 2 after 30 min of 70 degree tilt, acutely managed with application of CV Stim after failure of Sham Stim. Testing took place less than one month post-implantation. CBF (middle cerebral artery cerebral blood flow); dPdt (delta pressure/delta time—a measure of cardiac contractility). (**C**) Systolic BP changes. Systolic BP during supine rest (Baseline), symptomatic hypotension during tilt (Symptomatic), and steady state stimulation while tilted during three separate follow-up sessions for Participant 2. Stimulator patterns for Participants 1 and 2 are shown next to their respective graphs. Current (C) 5 mA, frequency (F) 50 Hz, pulse-width (PW) 350 μs were used for all electrode configurations.

During post-implantation tilt-table testing, Participant 1 was found to have no significant change in BP, and no significant effect of stimulation in the rostral direction (“CV Stim”) on BP was found (Fig. 5A). Her BP was monitored at all clinical visits, and stimulation was not found to affect the BP. During tilt-table testing, Participant 2 was found to have BP fall from a baseline in the range of 130/80 to 70s/40s mm Hg with symptoms of hypotension including nausea and dizziness. The eSCS applied with settings consistent with restoration of volitional movement, caudal direction (“Sham Stim”), was applied with no change in symptoms or recorded pressures. Stimulation was then applied rapidly in the rostral direction (“CV Stim”) up to the same amplitude with quick recovery of BP and resolution of symptoms (Fig. 5B).

**FIG. 6.**
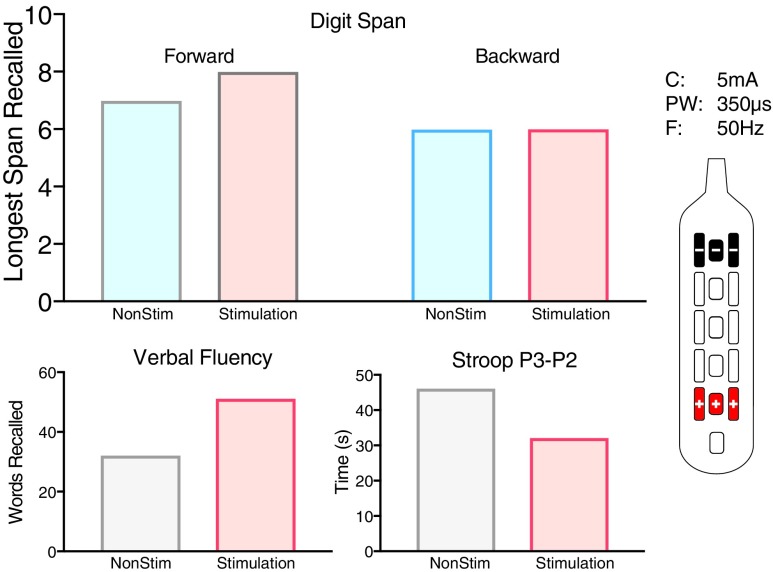
Cognitive changes during tilt and stim. Cognitive performance during orthostatic challenge test in Participant 2 on Digit span (top), Verbal Fluency (bottom left), and Stroop (bottom right) between hypotensive episode and CV stimulation demonstrating improved cognitive function. Lower scores on the Stroop test are representative of increased cognitive function. Control, no stimulation. Stimulation, stimulator pattern shown on the right. Current (C) = 5 mA, frequency (F) 50 Hz, pulse-width (PW) = 350 μs were used for all electrode configurations.

On comparing systolic BP (SBP) between lying flat before tilt (baseline), symptomatic hypotension immediately before stimulation (symptomatic), and during stimulation while remaining tilted (stimulation), significant differences were found during three separate testing sessions (Fig. 5C). Stimulation produced SBPs greater than during symptomatic hypotension while tilted (*p* < 0.001, *p* < 0.001, *p* < 0.001), and during two of the visits, SBP during stimulation was higher than while resting flat (*p* < 0.001, *p* < 0.001). In addition, during one trial, cognitive function measured during tilt testing without stimulation was reduced when compared with resting flat and after application of rostral (CV) stimulation while tilted (Fig. 6).

### Bowel and bladder function

Both participants had clinically significant bowel and bladder dysfunction at baseline. Participant 1 had worse bladder dysfunction compared with Participant 2, manifesting primarily as incontinence. Participant 2 had a NBDS of Moderate, while Participant 1 scored as Minor (Table 1). Over the course of five monthly follow-up visits, Participant 1 showed minor improvement in the Storage and Voiding subdomain of the NBSS. Participant 2 had an immediate large score improvement in the Incontinence subdomain with stable or unclear trends in all other subdomain values. Using rostral stimulation, Participant 2 reported the ability to void volitionally but incompletely with residual volumes (estimated 100 mL by post-void residual straight catheter). Early reports after implantation from Participant 2 included the restoration of bowel/bladder synergy.

**Table 1.** Bowel and Bladder Outcome Measure Scores per Visit

*ID #*	*Assessment*		*Visit Base*	*1*	*2*	*3*	*4*	*5*
1	NBDS		9	9	9	0	11	9
	NBSS	Con	4	4	4	5	4	4
		Inc	14	12	12	10	10	13
		Qua	1	2	2	2	2	2
		Sto	9	7	7	6	5	5
2	NBDS		11	8	8	0	14	9
	NBSS	Con	0	0	0	0	0	0
		Inc	7	0	0	0	0	0
		Qua	2	2	1	2	1	1
		Sto	3	3	2	0	2	4

NBDS, Neurogenic Bowel Dysfunction Score; NBSS, Neurogenic Bladder Symptom Scale; Con, consequences; Inc, incontinence; Qua, quality of life; Sto, storage.

Bowel dysfunction as measured by the NBDS reflected no change in the NBDS score in Participant 1 and remained stable during follow-up. Participant 2 experienced a slight worsening of bowel function after stimulation as measured by a change in the NBDS severity rating from Moderate to Severe. Despite this, Participant 2 reported a significant reduction in time spent on bowel regimen from 90 min to less than 30 min.

Participant 1 reported no change in sexual function. Participant 2 has consistently reported the return of the ability to achieve orgasm, absent since injury, during sexual intercourse while stimulation was on, or immediately after stimulation was stopped.

## Discussion

In this report, we corroborate that eSCS can restore volitional movement and muscle activity in participants after neurologically complete SCI. Importantly, our findings extend the generalizability of this finding to chronic SCI in females at older ages and longer intervals from injury than previously reported. Further, we demonstrate the ability of eSCS to improve cardiovascular function significantly during tilt-table testing with corresponding improvements in cerebral blood flow and cognitive performance. Surprisingly, while no changes in sensation were observed, our second participant reported restoration of sexual function with the ability to achieve orgasm. Last, both participants reported changes in bowel and bladder function, although the effect of these changes is indeterminate with this level of data. Participant 2 regained bowel/bladder synergy and volitional urination.

We chose to disseminate our novel discoveries at this early stage because the significant motor and autonomic findings have immediate implications that will impact perceptions about the generalizability of the benefit of eSCS for patients with SCI and influence a number of planned and ongoing clinical trials focused on eSCS after chronic SCI.

Both participants experienced immediate return of volitional movement with eSCS. Quantitative sEMG recordings during a modified BMCA support significant improvements in power within the first five visits compared with non-stimulation and after removal of evoked responses and evoked movements. The BMCA captures improvements in power of voluntary muscle activity, but, as it is performed in the supine position, it underestimates the recovery of muscles disadvantaged by gravity in the supine position such as those across the hip and knee.

The major limitation of sEMG, and by extension the BMCA, is noise, which is potentiated by stimulation artifact from eSCS. Conservatively subtracting the stimulation artifact and evoked motor activity and movements, as we have done, reduces sensitivity to subclinical muscle movements. The need for sensitive EMG measurement is questionable, however, because volitional movement is visible to the naked eye. Quantification of the strength and character of volitional movements is necessary to measure group and relative progress, but the accuracy required to detect subclinical changes is not required when functional movements remain the goal.

Observed movements were coarse and varied between follow-up visits based on parameter settings. Patients reported significant overall benefit from eSCS. While these early volitional movements were often large in amplitude, they were not functional movements. At higher intensities, evoked movements could be seen, although they remained under influence by each participant. This movement, which was non-cyclic and initiated by volitional cues without traditional local sensory input, is unlikely to be the result of central pattern generation demonstrated in animal models.^30–34^

The high frequency of the stimulation and the voluntary character of the movement that did not synchronize with the frequency or onset of stimulation also makes an SCS-evoked hypothesis unlikely.^35^ The observed movement was instead more similar to isolated, pattern-independent movements as observed in earlier motor complete spinal cord stimulator studies performed in patients with less chronic and less severe SCIs.^5,6^ The physiological explanation of this phenomenon remains unknown, but it is likely related to facilitation of weak volitional signals in residual descending motor fibers by reducing local hyperexcitability because of active eSCS.^14,36^ Follow-up experiments suggest that multimodal functional rehabilitation improves this effect.^8,9,14^ We believe that functional movements will require balanced restoration of coordinated muscles and that this may be possible with optimization and rehabilitation.^5,6,37,38^

Recovery of any volitional movement allows patients with complete SCI to participate in active rehabilitation exercises such as aided treadmill stepping, which have been shown to have positive long-term effects on walking capabilities.^39^ Insurance coverage for rehabilitation may be a result of prognostic factors for recovery. Injury severity is the greatest predictor of recovery, with motor complete injury having the least chance of recovery.^40^

The precise contribution of eSCS to the restoration of volitional movement had been unknown because previous reports applied both pre-habilitation and eSCS in all participants.^5–8^ Our study provides the first causal evidence that stimulation alone, as opposed to extensive pre-implant locomotor rehabilitation, is the primary factor in the restoration of volitional movement after SCI using eSCS. Although rehabilitation may play a significant role in recovery, especially in chronic SCI, these data raise questions about the relative importance and timing of intensive rehabilitation and eSCS. While physical therapy is clearly an important component of rehabilitation after SCI, the monetary costs and time investments required could preclude optimal efficacy. Our preliminary results indicate that eSCS, even in participants with long-standing injury, can immediately expand the capacity for volitional movement in participants with SCI. As such, eSCS may facilitate more significant rehabilitative therapy.

Significant differences between this protocol and previous work do prevent precise comparisons of functional recovery. Recruiting patients at more advanced ages and further from injury required significant safety considerations for injury, and it was decided to forgo attempts to stand until the effects of eSCS on patients provided sufficient evidence and data to design an intermediate protocol for improving bone density and muscle mass.

A recent similar study utilizing stand and step training in individuals with SCI documented one adverse event of a hip fracture in one of four participants, which was known to the investigators of this study before implementing that portion of the planned protocol.^10^ Multiple previous studies show an association between SCI and accelerated osteoporosis, which added to the context of the decision to forgo standing assessments.^41–47^ In a fashion similar to our remote data collection app, we hope a home rehabilitation program focused on improving bone density and atrophy during eSCS may result in candidacy for more advanced forms of therapy such as locomotor training in a safe and cost-effective manner.

While the restoration of volitional movement remains important to participants with chronic SCI, several studies report that restoration of autonomic function is a key component that is often overlooked.^16–18^ The autonomic nervous system regulates bladder, bowel, cardiovascular, and sexual function, and because of the loss of supraspinal connections after SCI, these systems are affected. Specifically, persons with SCI commonly experience the loss of voluntary bowel evacuation and reduced motility, which leads to prolonged bowel routine duration with manual evacuation.^48^ Sexual dysfunction is also prevalent, with the majority of individuals after SCI experiencing difficulties with erection, ejaculation, and the ability to experience orgasm.^49,50^ We found that sexual function, as characterized by restoration of orgasm, is possible with eSCS.

In addition, there is considerable cardiovascular dysfunction after SCI in participants with higher injuries, most commonly presenting as low resting BP and orthostatic hypotension (i.e., severe reductions in BP when assuming upright posture) or life-threatening episodes of autonomic dysreflexia.^51–53^ Reduced BP and associated reductions in cerebral perfusion often lead to episodes of pre-syncope, as well as subtle impairments in cognitive function.^51^ We have recently demonstrated that eSCS can modulate autonomic neurocircuitry responsible for cardiovascular control^16^; however, it had not been known whether other autonomic functions can be affected or if the restoration of supraspinal autonomic control is possible.

In the present work, we show evidence that there are significant changes in autonomic function with the application of eSCS. Quantitatively, the application of eSCS to restore cardiovascular function in Participant 2, who demonstrated cardiovascular dysautonomia on tilt and during ambulatory monitoring, provided immediate relief of symptomatic hypotension and reduced cognitive function. The effect was highly dependent on the direction of stimulation, requiring rostrally directed current, and was not effective during the use of settings typical for the restoration of volitional movement, as demonstrated in Figure 5. This key finding suggests that volitional movement and autonomic function are not optimized through the same stimulator settings and configuration, and that multisite stimulation (rostral and caudal) or broader coverage in general may be necessary for restoration of volitional movement and autonomic function.

Similarly, potential improvement of bladder scores, partial restoration of volitional urination in Participant 2, and some indication of normal bowel/bladder physiology with the return of synergy should warrant a particularly intense focus on quantitative measurement and optimization strategies because of the potential significance to the SCI community at large. Our findings extend recent published cases demonstrating that (1) epidural stimulation can immediately activate the cardiovascular system,^54^ (2) long-term stimulation may influence BP stability in persons with and without orthostatic hypotension,^20^ and (3) transcutaneous stimulation appears to improve lower urinary tract function.^55^

This work is limited in that there were only two subjects reported. As an early report to help guide eSCS study design, we anticipate that patient selection will be broader and significant effort placed on maximizing the recovery of autonomic function. We encountered other limitations during this study. Assessors and even sensory-complete participants can detect the presence of eSCS especially during changes to current. Therefore, blinding is not feasible for studies with eSCS. As a study focused on optimization (see online Supplement), the tested eSCS settings at each visit were chosen as the best by the participant's experience over each month from an objectively determined setting list provided by a Bayesian optimization. While informative of optimal parameters, this heterogeneity can be challenging for patients and providers.

These early findings from the E-STAND clinical trial indicate that eSCS may be beneficial for patients with minimal spinal cord axonal preservation on MRI, without undergoing intensive pre-implantation and locomotor training, more than 10 years from injury, and in the fifth and sixth decade of life. Moreover, we observed profound improvements in autonomic function, including restoration of cardiovascular regulation and sexual function, as well as promising changes in bowel and bladder function.

## Supplementary Material

Supplemental data

Supplemental data

Supplemental data

Supplemental data

Supplemental data

Supplemental data
