# Supplementary material for: Epidural Spinal Cord Stimulation Facilitates Immediate Restoration of Dormant Motor and Autonomic Supraspinal Pathways after Chronic Neurologically Complete Spinal Cord Injury
Source: J Neurotrauma. 2019 Jul 12;36(15):2325–36. doi: 10.1089/neu.2018.6006 (PMC6648195; doi:10.1089/neu.2018.6006)
Supplement: Supplemental data [file Supp_Fig1.pdf]

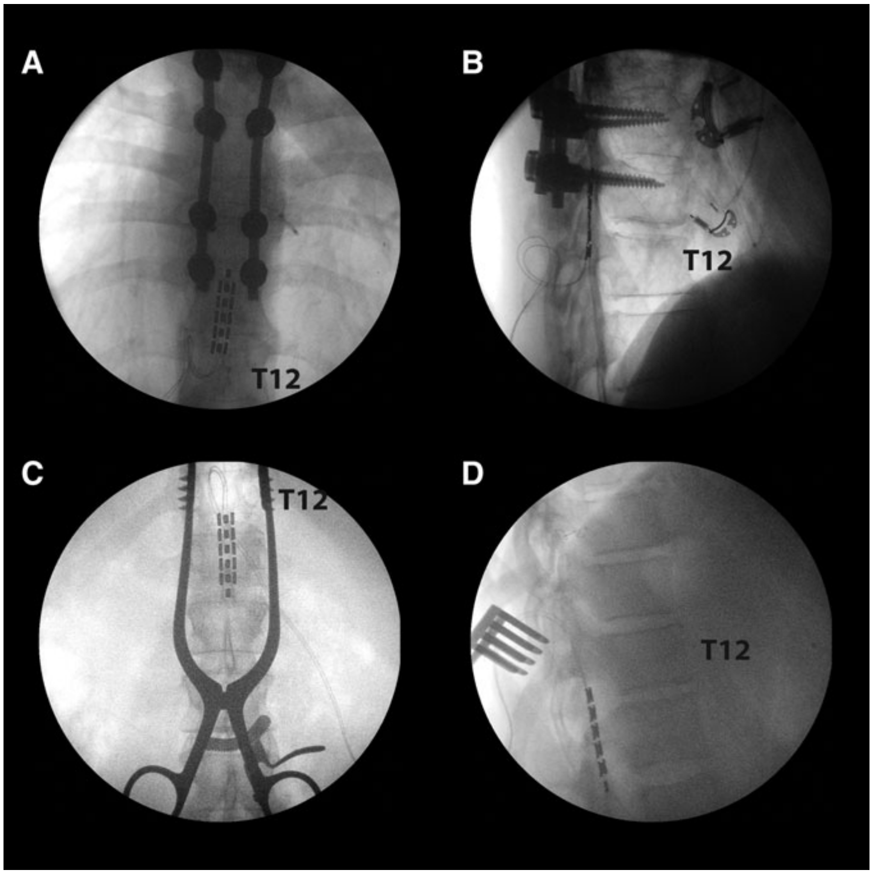

SUPPLEMENTARY FIG. S1. Final positioning of stimulator leads for Participant 1 (A: AP, B: Lateral) and Participant 2 (C: AP, D: Lateral) using intraoperative imaging. The T12 is marked for reference.
