# Supplementary material for: Epidural Spinal Cord Stimulation Facilitates Immediate Restoration of Dormant Motor and Autonomic Supraspinal Pathways after Chronic Neurologically Complete Spinal Cord Injury
Source: J Neurotrauma. 2019 Jul 12;36(15):2325–36. doi: 10.1089/neu.2018.6006 (PMC6648195; doi:10.1089/neu.2018.6006)
Supplement: Supplemental data [file Supp_Fig2.pdf]

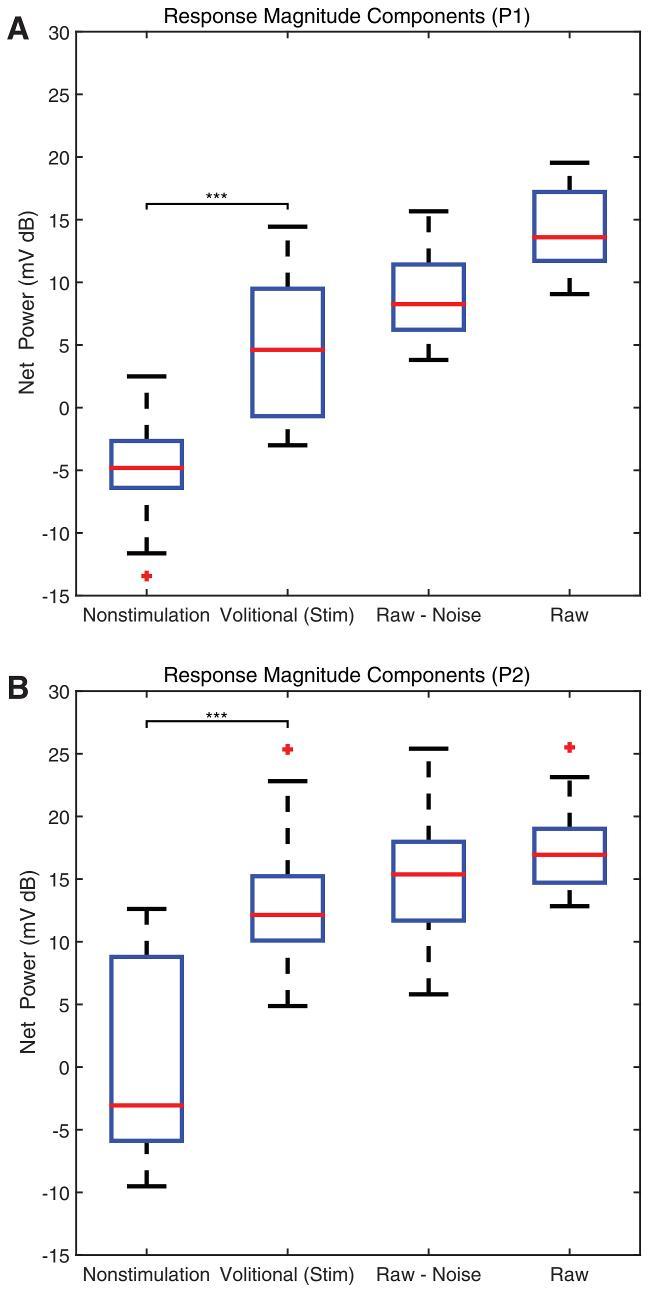

SUPPLEMENTARY FIG. S2. Response magnitude components. For each patient, the raw pooled root-mean-square power (Raw), correction after removal of power from noise (Raw - Noise), correction after removal of power generated during rest, which would include spinal cord stimulation (SCS)-evoked responses (Volitional [stim]), and the corrected power calculated in the same way when SCS is off (Non-stimulation). **A**: Participant 1, **B**: Participant 2. Asterisks denote statistical significance: $*p<0.05$, $**p<0.01$, $***p<0.001$.
