# Supplementary material for: Epidural Spinal Cord Stimulation Facilitates Immediate Restoration of Dormant Motor and Autonomic Supraspinal Pathways after Chronic Neurologically Complete Spinal Cord Injury
Source: J Neurotrauma. 2019 Jul 12;36(15):2325–36. doi: 10.1089/neu.2018.6006 (PMC6648195; doi:10.1089/neu.2018.6006)
Supplement: Supplemental data [file Supp_Text.pdf]

Supplementary Text

Response Magnitude of the Brain Motor Control Assessment

The voluntary portion of the Brain Motor Control Assessment (BMCA) is composed of six flexion and extension maneuvers sequentially repeated three times. The maneuvers include two simple tasks: (1) hip flexion/knee flexion followed by extension and (2) ankle flexion followed by extension performed bilaterally, then unilaterally (Right then Left). Each maneuver is repeated three times sequentially for the purposes of averaging. Ideally, the first 5 sec of each task primarily is flexion while the second 5 sec primarily is extension. Accurately timing each phase of the task intrinsically does assume that there exists sufficient control and strength to time movements appropriately, which was found to be unreliable in these participants. The entire 10 seconds was used to evaluate power generated from movement.

Surface EMG (sEMG) is limited by extensive noise including movement artifacts and stimulation artifact. The raw sEMG is filtered with a low-pass Butterworth filter ($N=4$); the root-mean-square (RMS) power is calculated for each channel. The three trials are averaged together, resulting in six powers for 10 muscle groups (16 total, including abdominal, intercostal, and paraspinal).

The original report of the Voluntary Response Index removed the baseline activity by subtracting the RMS power present during the 1 sec preceding the first volitional task, but we found this to not represent accurately the baseline power over an entire set of tasks. For a more accurate estimate of the baseline power, we calculated the root-median-squared power during the last 5 sec of each channel of each BMCA task. The median is less sensitive to deviations from movement. This baseline was subtracted from the trial averages.

Further, the BMCA also has a relaxation time (5 min) toward the beginning of recording. Stimulation is either on or off for the entire BMCA, and the BMCA is completed in both conditions at each follow-up appointment. To account for possible evoked responses, RMS power was calculated during relaxation for 30 windows of 10 sec after removing the baseline power. The median power for each channel was removed from each task power to result in the Nonstimulation and Volitional (Stim) results in Figure 3.

Optimization

Spinal cord stimulation (SCS) systems provide flexible spatial configurations of electrodes to augment the spatial pattern of electrical current, and settings can be used to change the frequency, pulse width, and current of unique settings to optimize SCS for volitional movement and autonomic function. The broad number of permutations provides a unique challenge to find the optimal stimulation parameters for restoration of function. A nearly infinite number of settings and spatial configurations (>4 quadrillion) are possible.

Efficient strategies for evaluating numerous parameters require coordinated selection of complementary settings and vast quantities of data. In addition, optimized settings and configurations for one outcome measure may have no resemblance to those for a different outcome measures and may change over time.[1] Generalizability of optimal settings remains unknown across patients and outcomes. Last, rapid, quantifiable measurements are not obtained easily for all outcomes such as pain or autonomic function (bowel or bladder).

Because of the dramatic differences in functional changes between even slight changes in stimulator settings, there is great need for optimization.[1–3] To simplify electrode configuration, we have chosen to stimulate broadly, only making small adjustments to the symmetry. An overarching aim of E-STAND focuses on determining the optimal stimulation parameters given this simple configuration of the electrodes. Through the use of remotely collected patient surveys and a simple app-based accelerometer task (described below), we have developed an optimization strategy that identifies the best setting (of eight assigned) during the course of one month between follow-up visits.

Patients receive eight settings that span frequency and pulse width space each month. Patients are assigned a specific sequence to obtain pairwise comparisons between settings throughout the month. Quantitative accelerometry is obtained during a 10 min task that patients complete at home where they are instructed to move each side when cued. Accelerometers are placed on the dorsum of each foot. The tablet application provides a visual cue that signals the patient to flex the hip and extend the leg on either the right or left side.

Patients are asked to compare their preference for settings as they evaluate one setting each day. Response surfaces are generated for quantitative and subjective data. An adaptive Bayesian approach evaluates the response surfaces to identify the next set of settings to (1) refine around optimal peaks identified, (2) provide redundancy, and (3) search untested parameter space. As the trial continues, participants obtain increasingly optimized parameters for this broad stimulation. The first five visits for these first two participants evaluated the frequency space primarily. Details about the optimization and validation of the approach will be discussed further in a subsequent publication.

Early Trial Challenges

While originally designed as a blinded assessment, both patients know immediately when stimulation is on or off at any significant level despite the lack of sensation, confounding attempts to blind through randomized blocking. The current approach to the spatial configuration of current is based on facilitating broad stimulation. Highly specific stimulation can restore function in specific muscle groups, and we attempted to recruit the maximum number of muscle groups with the knowledge that precise current delivery patterns to ensure individualized nerve root thresholds are met will be possible in future devices.

Supplementary Material References

1. Rejc, E., Angeli, C.A., Bryant, N., and Harkema, S.J. (2017). Effects of stand and step training with epidural stimulation on motor function for standing in chronic complete paraplegics. J. Neurotrauma 34, 1787–1802.
2. Rejc, E., Angeli, C.A., Atkinson, D., and Harkema, S.J. (2017). Motor recovery after activity-based training with spinal cord epidural stimulation in a chronic motor complete paraplegic. Sci. Rep. 7, 13476.
3. Aslan, S.C., Legg Ditterline, B.E., Park, M.C., Angeli, C.A., Rejc, E., Chen, Y., Ovechkin, A.V., Krassioukov, A.V., and Harkema, S.J. (2018). Epidural stimulation of lumbosacral modulates arterial blood pressure in individuals with spinal cord injury-induced cardiovascular deficits. Front. Physiol. 9, 565.
