# Supplementary material for: Epidural Spinal Cord Stimulation Facilitates Immediate Restoration of Dormant Motor and Autonomic Supraspinal Pathways after Chronic Neurologically Complete Spinal Cord Injury
Source: J Neurotrauma. 2019 Jul 12;36(15):2325–36. doi: 10.1089/neu.2018.6006 (PMC6648195; doi:10.1089/neu.2018.6006)
Supplement: Supplemental data [file Supp_Fig3.pdf]

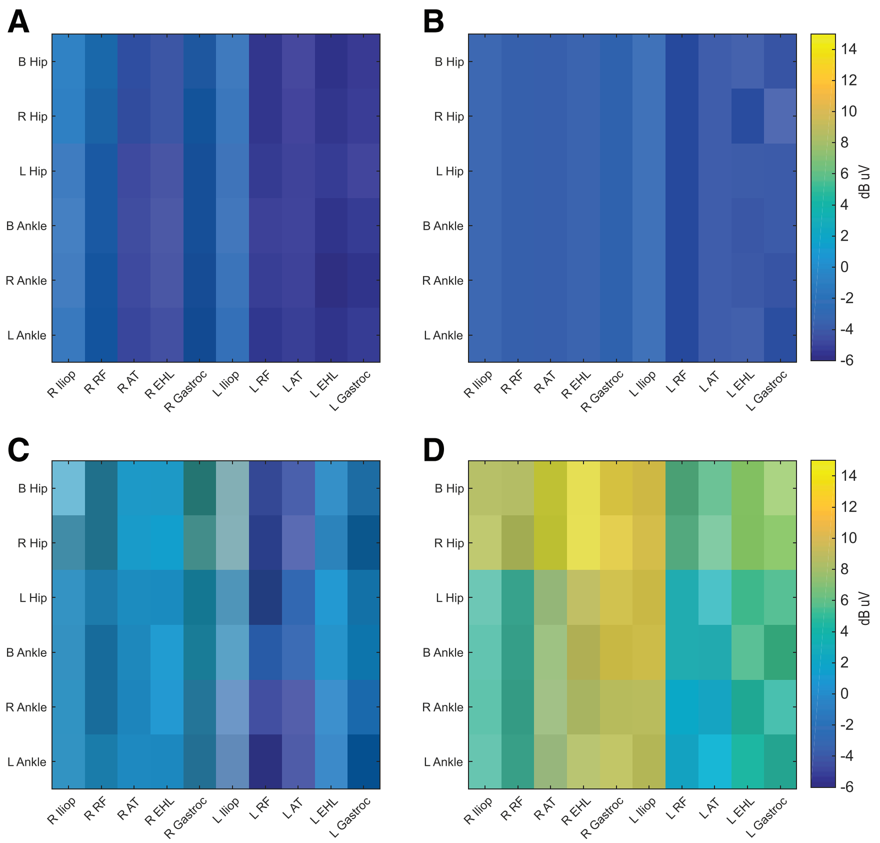

SUPPLEMENTARY FIG. S3. Muscle power and tasks. Surface electromyography power averaged over follow-up visits during Brain Motor Control Assessment volitional tasks at Baseline/Non-stimulation (top) and during Stimulation (bottom) for Participant 1 (left) and Participant 2 (right). Refer to main article Figure 3 for stimulator pattern and settings information.
